# Machine Learning in Membrane Design: From Property Prediction to AI-Guided Optimization

**DOI:** 10.1021/acs.nanolett.3c05137

**Published:** 2024-03-04

**Authors:** Zhonglin Cao, Omid Barati Farimani, Janghoon Ock, Amir Barati Farimani

**Affiliations:** † Department of Mechanical Engineering, Carnegie Mellon University, Pittsburgh Pennsylvania 15213, United States; ‡ Department of Chemical Engineering, Carnegie Mellon University, Pittsburgh Pennsylvania 15213, United States; § Machine Learning Department, Carnegie Mellon University, Pittsburgh Pennsylvania 15213, United States

**Keywords:** machine learning, membrane design, 2D materials, polymeric membranes, nanopore, data-driven design

## Abstract

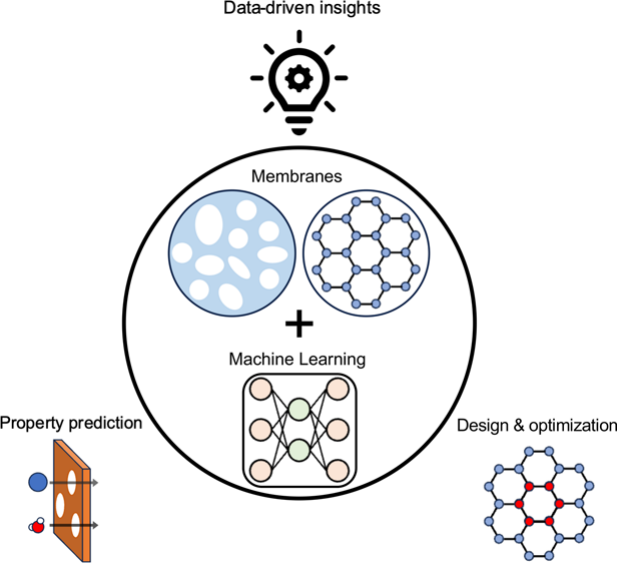

Porous membranes, either polymeric or two-dimensional materials, have been extensively studied because of their outstanding performance in many applications such as water filtration. Recently, inspired by the significant success of machine learning (ML) in many areas of scientific discovery, researchers have started to tackle the problem in the field of membrane design using data-driven ML tools. In this Mini Review, we summarize research efforts on three types of applications of machine learning in membrane design, including (1) membrane property prediction using ML, (2) gaining physical insight and drawing quantitative relationships between membrane properties and performance using explainable artificial intelligence, and (3) ML-guided design, optimization, or virtual screening of membranes. On top of the review of previous research, we discuss the challenges associated with applying ML for membrane design and potential future directions.

Porous membranes are critical to various real world applications. Among all types of porous membranes, a polymeric membrane is the one being widely adopted for industrial water filtration, including nanofiltration (NF) and reverse osmosis (RO), as a substitution for other water treatment methods like distillation because of lower energy consumption and cost.^1^ Another important application of polymeric membranes is gas separation such as carbon dioxide removal.^2^ In the recent decade, two-dimensional (2D) materials, including graphene, have emerged as the next-generation membrane materials. The outstanding mechanical strength combined with the subnanometer thickness of 2D materials gives them potential of being the future upgrade of polymeric membranes.^3^ For example, computational and experimental efforts have shown that 2D materials with nanopores are more energy-efficient in RO desalination compared with polymeric membranes.^4,5^ Behind the successful application of porous membranes, the discovery and design process of both polymeric and 2D membranes are based mostly on traditional trial-and-error experimental methods, which are time-consuming and expensive. For polymeric membranes, the process of fabrication and synthesis can significantly affect their properties.^6^ However, the large search space constituted by the selection of polymers, solvent, and additives renders traditional experimental methods inefficient in discovering new polymeric membranes.^6^ One example of such inefficiency is the marginal RO performance improvement of polymeric membranes since 1990.^7^ For 2D material membranes, their performances depend not only on the type of materials^8,9^ but also on the size and geometry of the artificial nanopores.^10,11^

Due to the higher cost of synthesis and the sheer amount of possible nanopore geometries on 2D materials,^12^ exhaustive experimental methods fall short again in efficiently discovering or searching the optimal membrane for desired properties. Furthermore, physics-based computational approaches, such as molecular dynamics simulation, face challenges in conducting exhaustive searches due to their computational intensity, which limits the assessment of a wide range of candidate membranes. As more experimental or computational simulation data on polymeric and 2D membranes have accumulated, researchers recently started to harness the power of data-driven machine learning (ML) tools to overcome the aforementioned obstacle. By using ML models for rapid screening and optimization, researchers can efficiently evaluate a wide array of membranes, substantially reducing the time and resources required for experimental synthesis and computational assessments. To advance further, these ML models offer valuable insights into the characteristics of membranes and support inverse design processes tailored to the desired target properties. This methodology greatly aids in pinpointing promising materials for specific uses, thereby extending the frontiers of chemistry and materials science.

The advancement of ML has driven revolutionary changes in many areas of scientific discovery. The most common scenarios of applying ML for science is to use discriminative ML models, which focus on distinguishing between different outcomes or classes, to predict the desired properties of a given input (e.g., the band gap of a crystal^13^ and the adsorption energy of a catalyst^14,15^). ML models trained in a supervised manner can detect the nonlinear relationship between features of the input and the corresponding properties, leading to accurate and fast predictions. With the exponential growth of experimental and computationally generated data sets, ML models have become a powerful tool that allows researchers to rapidly explore the chemical and material space.

The significant achievements of machine learning (ML) models in diverse chemical and material domains—including inorganic crystals, organic molecules, and polymeric materials—underscore their broad applicability and potential for extension into many other subset domains. Given this, ML has recently been employed for the purpose of membrane design. For example, ML models have been employed to predict gas permeability in polymeric membranes, as demonstrated by Hasnaoui et al.^16^ The comprehensive review by Brown et al.^17^ provides an insightful overview of various machine learning and deep learning model architectures, basic principles in model setup, and their corresponding application in material discovery and design. In this paper, we review the research progress of the three major use cases (Figure 1) of ML in membrane design: (1) ML property prediction of polymeric or 2D material membranes, where the predicted properties include not only the performance in certain applications such as the water permeation rate in water filtration but also physical properties of the membranes such as the synthesizability or stability, (2) the use of explainable AI (XAI) to understand the physical relationship between features of membranes and their performance in applications by quantifying the feature importance, and (3) data-driven design of membranes by either ML-accelerated virtual screening or ML-guided membrane optimization. We conclude the Mini Review with a discussion about the current challenges associated with ML in membrane design and the outlook for future directions in this domain.

**Figure 1:**
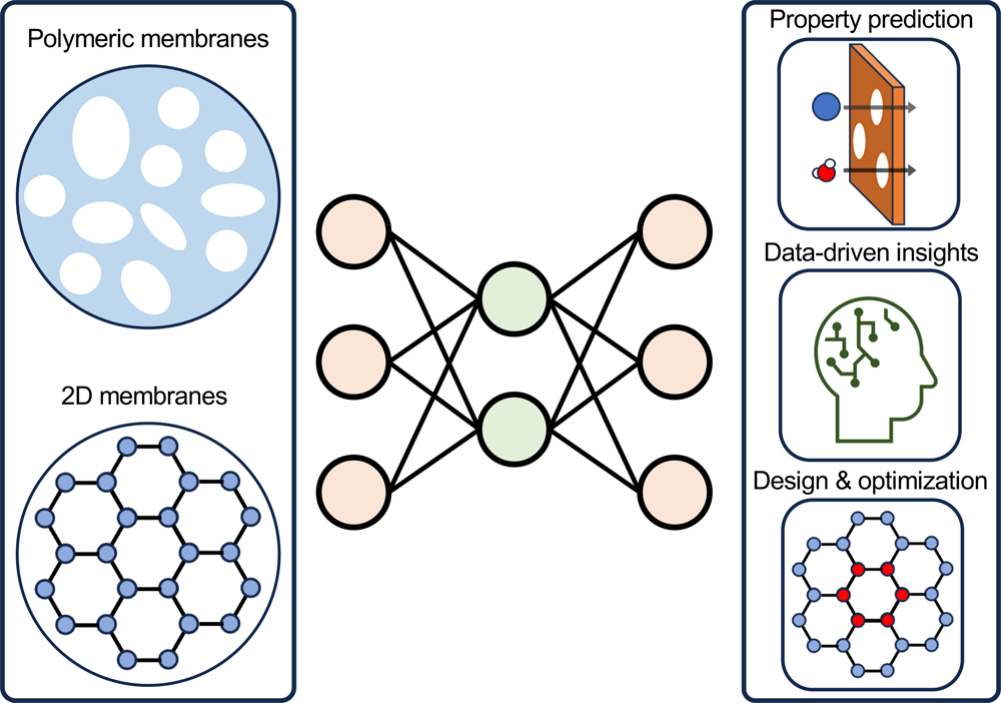
Schematics of applications of machine learning in membrane design.

## ML for Membrane Property Prediction

ML models are often used as surrogates for experiments or other traditional computational methods for membrane property prediction. After training on a labeled data set, ML models are capable of making an instant inference on the desired properties of membranes. Normally, in order to train ML models to make predictions, featurization of the membrane data is necessary. Featurization (fingerprinting) is a process that represents membranes with numerical values that can be used as inputs to the ML models. The featurization of membranes varies by the type of the membranes (polymeric or 2D materials) and can affect the choice of ML models for the prediction task. In this section, we review the recent research on ML membrane property prediction categorized by membrane type with a focus on the featurization methods and the corresponding ML model selection.

**Figure 2:**
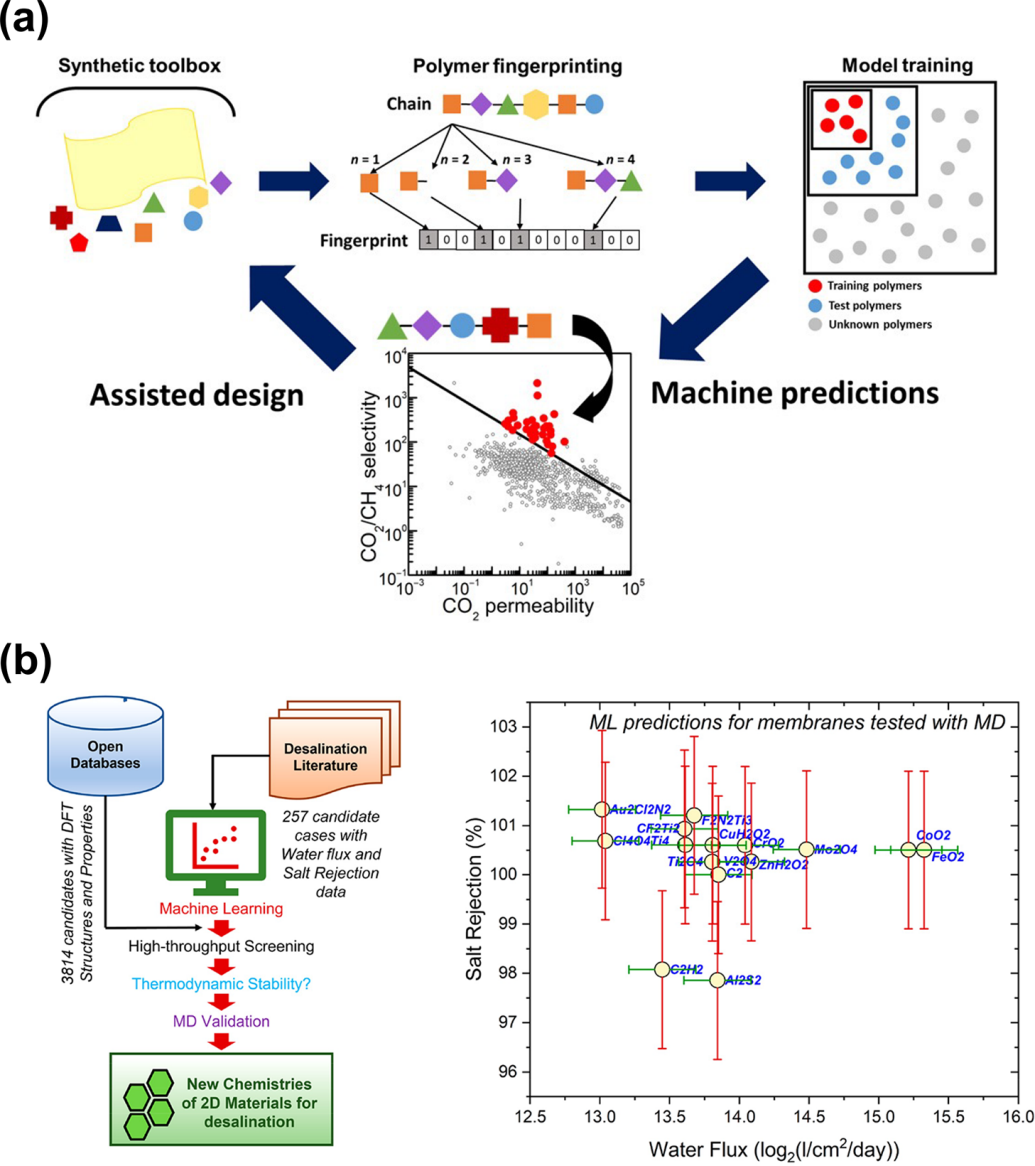
Integrating machine learning in membrane technology: gas permeability and water desalination performance prediction. (a) Machine learning workflow to predict gas permeability of polymeric membranes in Barnett et al.^18^ Polymers are transformed into binary fingerprints for training, after which high-performing sets are pinpointed through predictions to facilitate ML-assisted design. From ref (18). Copyright The Authors, some rights reserved; exclusive licensee AAAS. Distributed under a CC BY-NC4.0 license http://creativecommons.org/licenses/by-nc/4.0/. Reprinted with permission from AAAS. (b) Machine learning workflow of predicting reverse osmosis water desalination performance of 2D materials membranes proposed by Priya et al.^9^ (left), and the comparison between machine learning prediction and molecular dynamics simulation results (right). Prediction errors are shown by the error bars (green for water fluxes and red for salt rejection rates). Reproduced with permission from ref (9). Copyright 2022 American Chemical Society.

Polymeric membranes are very effective in the task of gas separation. Accurate ML prediction of gas selectivity or permeability of polymeric membranes can significantly accelerate the screening for best candidates. In a work by Hasnaoui et al.,^16^ a neural network is used to predict the gas permeability of polymeric membranes. The featurization of the polymer in this work is based on the group contribution approach, which regards a polymer as combinations of multiple repeating units. On a experiment data set with 147 polymers, the neural network was shown to achieve correlation factors (*R*) of 0.999, 0.999, 0.984, and 0.999 for N_2_, O_2_, CO_2_, and CH_4_ permeability prediction, respectively. Barnett et al.^18^ trained a Gaussian Process Regression (GPR) model on a data set consisting of approximately 700 polymeric membranes with experimentally measured permeabilities of different gases (Figure 2a). After that, the GPR model was used to make predictions on more than 11000 polymers. The ones predicted to exceed the previous upper bound were experimentally validated. Using this screening process guided by ML prediction, they were able to find 2 polymers that surpassed the then state-of-the-art in CO_2_/CH_4_ separation. In this work, the polymers are featurized as hashed fingerprints that contain both cheminformatics of each monomer and the topological information.

Water treatment is another critical application of polymeric membranes. The salt rejection and the water flux are the two major desalination performance evaluation metrics of membranes to be predicted by ML. In the work of Zhang et al.,^19^ the authors proposed a featurization strategy of polyamide membranes for rejection and flux prediction. The features of the membranes included the type of supporting membrane, chemical structure (Cartesian atom coordinates calculated by density functional theory), concentration of the monomer of polyamide membrane, and operation pressure of the nanofiltration. Moreover, the authors proposed augmenting the monomer structure using vibration. Overall, trained on only 100 data points, the proposed featurization method with a neural network model was shown to achieve a correlation coefficient of 0.8 and mean relative error of 5% when predicting flux and rejection of unseen membranes. For ultrafiltration water treatment using nanocomposite membranes, Fetanat et al.^20^ designed a software platform that incorporated a neural network with a graphical user interface to conveniently predict the solute rejection, flux recovery, and pure water flux. In this work, the input to neural network is nanocomposite membrane information including polymer type, polymer concentration, filler concentration, average filler size, solvent type, solvent concentration, and contact angle used in membranes. Pervaporation is a technology for liquid mixture separation by using membranes. Wang et al.^21^ trained a gradient boosting regressor using 681 data samples (16 different polymers and 6 organic solvents) collected from the literature. Two featurization methods were benchmarked including one called *bag-of-fragments*,^22^ which encodes the molecular information on the polymer and solvent. Using the trained ML model, the authors screened approximately 1 million hypothetical polymers for pervaporation separation of a water/ethanol mixture. Ten candidates were identified to be synthesizable and surpass experimental samples in performance.

2D material membranes are versatile, as they have been demonstrated to have outstanding performance in applications such as water desalination.^8^ For many applications, creating artificial nanopores on the 2D material membranes is necessary to allow molecule/atom separation and translocation. The high synthesis cost^23^ as well as the precise process required for nanopore creation^24^ can be prohibitive for rapid screening of 2D material membranes. ML models are utilized as data-driven surrogates to experimental methods for predicting properties of the 2D membranes. In the work of Priya et al.,^9^ a ML pipeline was built to predict the water flux and ion rejection rate of nanoporous 2D membranes in RO water desalination (Figure 2b). The 2D materials were represented by 44 features, including structure, chemistry, and atomic partial charges of the pore and membrane, that were selected based on domain knowledge. Those features were used as input to a decision-tree based XGBoost^25^ model for prediction. By the feature importance calculated by the XGBoost model, the maximum positive and negative charge in the membrane and the membrane atomic number were the most important membrane-related features that determined the flux and rejection prediction. After screening through 3814 2D materials from the literature and validating the result using molecular dynamics simulations, the authors revealed that having transition metals at the pore could improve the ion rejection rate of the membrane. They also found candidates such as FeO_2_ could reach ∼4 times higher water flux than graphene. Besides the material of the membrane, the geometry of nanopores was also shown to influence the membrane performance in RO water desalination.^10^ However, the number of possible geometry of nanopores is astronomical (i.e., theoretically 11.7 million when pore size is 20 atoms on a graphene lattice).^12,26^ In the work of Wang et al.,^27^ a convolutional neural network (ResNet)^28^ was used to predict the water flux and ion rejection rate of graphene nanopores. Since the desalination conditions and the membrane material were fixed, the model takes only an image of the nanoporous graphene membrane as input and automatically extracts geometrical features to make accurate predictions. Such a method enabled fast evaluation of graphene nanopores for water desalination. To study the formulation probability and time of graphene nanopores, Sheshanarayana and Ananth^29^ trained a CatBoost^30^ model using data generated by kinetic Monte Carlo simulation. The model achieved *R*^2^ values of 0.97 and 0.95 for nanopore probabilities and formation time prediction, respectively. It enabled quantification of the ease of formation of a given nanopore shape in graphene via silicon-catalyzed electron-beam etching.

## Data-Driven Understanding of Membranes Using XAI

Explainable AI, or XAI,^31^ methods have been developed for the purpose of justifying the prediction of ML models and extracting knowledge from models in a data-driven manner.^32^ This is particularly significant, given that ML models typically function as black boxes. When being applied to membrane design, XAI methods are exceptionally useful, as they can help to quantify correlations between features of membranes and desired properties. The key features identified by XAI can be validated using a physics-based approach, enabling the rigorous application of ML in membrane design. This provides a means to manipulate the features to achieve targeted properties. In this section, we introduce several XAI tools and their use cases in membrane design.

**Figure 3:**
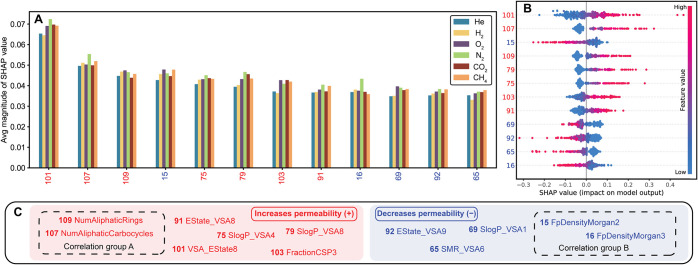
Assessing feature impact on gas permeability prediction using SHAP values. The SHAP value is used to quantify the impact of different features on the model’s prediction of gas permeability (subpanels (A) and (B)). (C) shows the mapping of feature index to the name of the feature for better readability. From ref (33). Copyright The Authors, some rights reserved; exclusive licensee AAAS. Distributed under a CC BY-NC4.0 license http://creativecommons.org/licenses/by-nc/4.0/. Reprinted with permission from AAAS.

The Shapley value^34^ is the foundation of many XAI methods that attribute the prediction of ML model to input features. The *SHapley Additive exPlanations*(35) (SHAP) package is a unified framework built on the basis of Shapley value to interpret ML predictions. After a ML model is trained to predict membrane properties in the supervised manner, the SHAP package can be used to quantitatively analyze the correlation between the properties and membrane features.^36^ In the work of Yang et al.^33^ (Figure 3), the SHAP value was used to interpret the multitask gas permeability prediction by a random forest (RF) and deep neural network (DNN) ensemble model. The models were trained to predict the permeability of 6 different gases (He, H_2_, O_2_, N_2_, CO_2_, and CH_4_) through polymeric membranes. The benchmark on test sets showed that the DNN ensemble with the Morgan fingerprint with frequency achieved the highest *R*^2^ score for all gases. The analysis showed that the number of aliphatic cycles had a significantly high positive impact on the CH_4_ permeability because of its high SHAP value. The fingerprint density was also shown to have a negative impact on the CH_4_ permeability. In general, the SHAP analysis, consistent with experimental results, showed that repeating units with more nonaromatic rings allowed for larger free-volume elements and lower densities, and thereby higher gas permeabilities. In another work by Jeong et al.,^37^ SHAP analysis was used to discover the correlation between features of RO and NF membranes with the ion rejection rate. SHAP analyses were performed on decision tree based models after they were trained to predict the ion rejection rate of polymeric membranes. The SHAP analysis identified that the electrostatic interactions (charge product) were more critical in determining anion rejection than cation rejection, whereas the hydrated radius (related to size exclusion and ion dehydration) contributed more to the prediction of cation rejection. A SHAP analysis demonstrated the high importance of molecular weight cutoff (MWCO) and hydrated radius to model prediction, correctly reflecting how membrane pore size and ion size regulated salt/ion permeation through RO and NF membranes. Overall, SHAP is a powerful XAI tool that can be used to provide data-driven insight into the membrane design process.

Besides SHAP analysis, other machine learning methods were used to discover or explain the physical properties of membranes. Ritt et al.^38^ used machine learning as a statistical analysis tool to identify the most important features for predicting thermodynamic barriers of ion transport through polymeric membranes. The feature pool contained 126 features obtained from DFT calculation, cheminformatics, and a literature search. Linear regression models were trained to predict enthalpic (Δ*H*^⧧^), entropic (*T*Δ*S*^⧧^), or the free energy barrier (Δ*G*^⧧^) for anion permeation using a subset of features selected by a recursive feature addition method. The contribution of features to the prediction was determined and ranked by the absolute coefficient in the linear regression model. The relationship between the pore and the ion electrical properties was found to be critically important to the free energy barrier of ion permeation. Such a finding could be extended to polymeric membrane design. In another work by Yeo et al.,^39^ the feature importance in the gradient boosting tree model was used to identify key parameters of thin film nanocomposite membranes for better RO desalination performance. After the model was trained on a data set collected from the published literature, a feature importance analysis suggested that porous nanoparticles could perform better than nonporous ones. Loading, pore size, and hydrophilicity were also identified as primary factors that influenced water permeability and salt passage within the membrane. The two works mentioned above demonstrate that model-dependent methods (e.g., coefficient in linear regression and feature importance in tree-based models) are useful to understand the performance and properties of membranes.

## ML-Assisted Membrane Screening and Optimization

Automatic and data-driven screening and optimization of membranes are the third application of ML in membrane design. In a trial-and-error membrane screening or design process, researchers need to manually select/modify membranes, evaluate the properties of the new samples, and then determine whether the selection/modification is desirable. With the help of predictive ML models, researchers are now able to build pipelines to automate such a process. In this section, we present several works that cover both ML-assisted membrane screening and optimization.

Bayesian optimization (BO) is a method that uses an active search to optimize an intractable function *f*(*x*) given an input *x*. BO has recently extended its application to membrane design. In a work by Gao et al.,^40^ ML-based Bayesian optimization was used to identify the optimal combinations of monomers and their fabrication conditions for water desalination. Tree-based ML models were trained to predict water permeability and ion rejection of membranes, given the fingerprint of membrane monomer and fabrication conditions. BO was then used to inversely identify sets of monomer/fabrication condition combinations that could potentially break the known upper bound of desalination performance. Eight of the top 10 combinations were fabricated and experimentally validated to have outstanding performance in desalination, which further demonstrated the effectiveness of BO in designing membranes.

**Figure 4:**
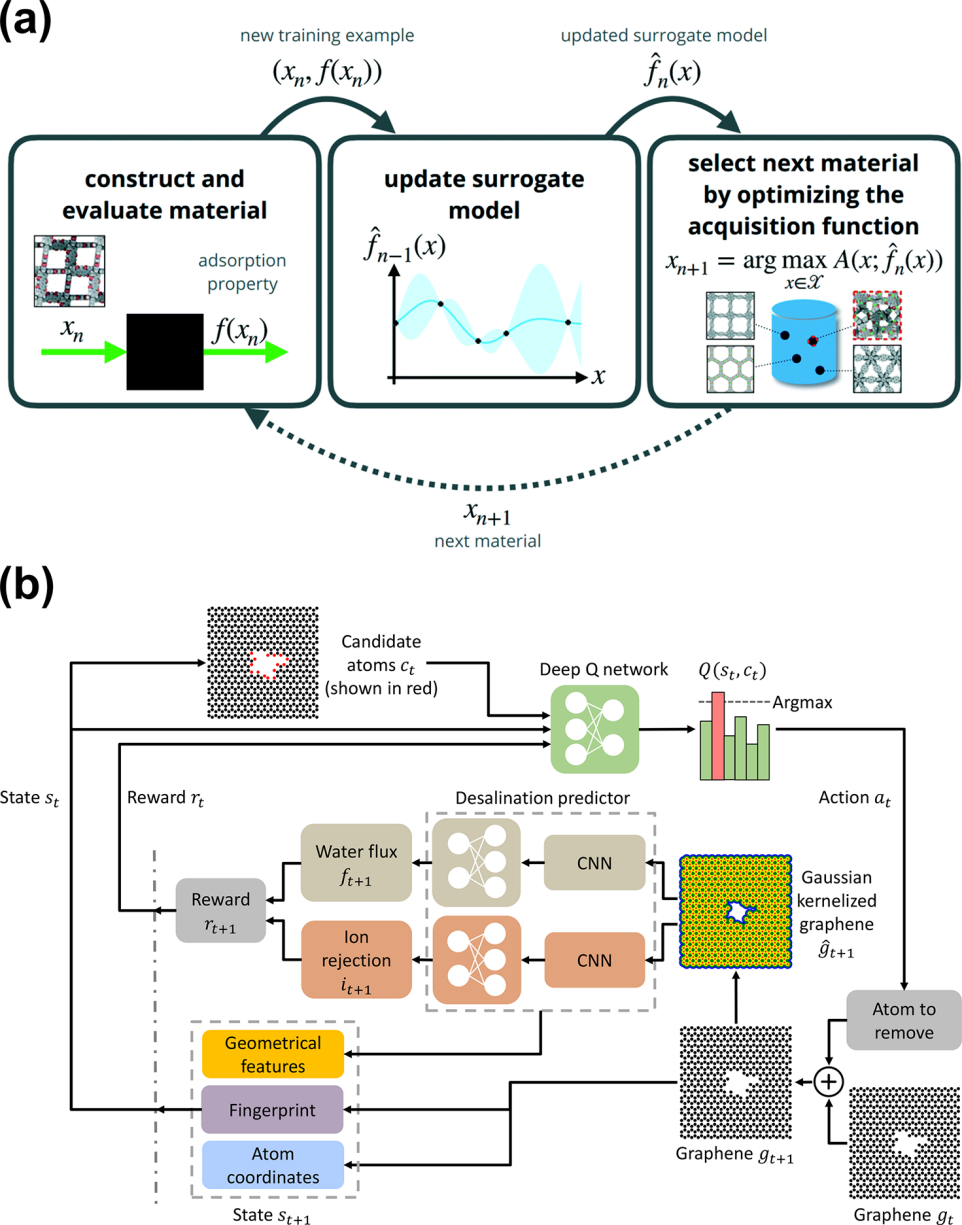
Applications of Bayesian optimization and deep reinforcement learning in nanoporous material. (a) Schematics showing the application of Bayesian optimization in screening nanoporous materials. The process involves a cycle of measuring material properties, updating a surrogate model of the objective function, and choosing the next material for testing to optimize a black-box function. Reproduced with permission from ref (41). Copyright 2021 Royal Society of Chemistry. (b) Deep reinforcement learning framework proposed by Wang et al.^27^ to automatic optimize graphene nanopore geometry for reverse osmosis desalination. A convolutional neural network is used to predict water flux and ion rejection rate of nanopores. Reprinted with permission under a Creative Commons CC BY4.0 license https://creativecommons.org/licenses/by/4.0/ from ref (27). Copyright 2021 The Authors.

Aside from polymeric membranes, BO is also useful in screening nanoporous materials such as covalent–organic frameworks (COFs) and metal–organic frameworks (MOFs). COFs and MOFs are groups of porous materials (some are in the form of membranes^42^) that are prevailing in gas separation.^43^ The modular structure of COFs and MOFs allows for an almost unlimited variety of building block combinations,^43^ rendering the virtual screening a exhausting task. Deshwal et al.^41^ employed BO to rapidly screen COFs for methane deliverable capacity (Figure 4a). A Gaussian Process (GP) model was used as the surrogate model, an approach to approximate complex simulations to predict outcomes in a data-driven manner in BO as it could model the probabilistic relationship between features of a COF and its property. From a database consisting of 70000 COFs, BO iteratively selected COFs that optimized the acquisition function, obtained their methane deliverable capacity using grand-canonical Monte Carlo (GCMC) simulation, and retrained the surrogate model using the enlarged training data set. BO was demonstrated to identify the top 100 COFs in the database after simulating only 139 COFs, which was highly sample efficient. Other deep learning based methods have also been used for COF/MOF virtual screening. Wang et al.^44^ proposed a top-down virtual screening method combining a crystal graph convolutional neural network^13^ and GCMC simulation. Using such a method, they screened the hypothetical MOF^43^ data set and identified the top 4 candidates in methane adsorption. With the emergence of more advanced transformer-based deep learning models,^45,46^ the virtual screening of MOFs/COFs can proceed with better prediction accuracy and higher sample efficiency.

Nanopore optimization on membranes is another field where ML excels besides membrane material screening. On 2D membranes such as graphene, there is a myriad of variations in nanopore geometry^12^ and the geometries determine the membrane performance in applications like water desalination.^10,11^ Optimizing the nanopore geometry for desired performance can be a Herculean task because of the sheer size of the search space. To tackle such a problem, Wang et al.^27^ designed a deep reinforcement learning (DRL) framework to optimize the graphene nanopore geometry for RO water desalination (Figure 4b). In the DRL framework, an agent modeled by a neural network was trained by iteratively removing carbon atoms from the center of a graphene membrane. The geometrical information on nanoporous graphene membranes was formatted as images. A ResNet^28^ convolutional neural network was used to take the images as input and predicted the water flux and ion rejection rate of nanoporous graphene membranes. The atom removal action of the agent was judged by the reward signal that balanced the trade-off between water flux and ion rejection rate. After training, the DRL agent discovered a geometry pattern that was validated by molecular dynamics simulation to reject 8% more ions than circular nanopores while maintaining the same water flux. The DRL framework could be easily extended to optimize nanopore geometry for other applications or on other 2D materials.

## Challenges and Outlook

In this Mini Review, we have discussed the application of ML in membrane design from membrane property prediction and XAI for a data-driven understanding of membranes to ML-assisted screening and optimization. ML in membrane design is still a rapidly developing field, as many related works have been published within the past 5 years. With the demonstrated effectiveness of ML in membrane design, research about this topic will witness further growth as more powerful ML models are developed and more experimental data of membranes are published. Additionally, the progress in XAI will bring a data-driven understanding of the relationship between membrane features and desired properties. From our perspective, there are three major challenges and opportunities for the future.

The first and most important one is the lack of large and consistent data sets, especially for polymeric membranes. ML models, especially deep learning models, can be very data-hungry when fitting high-dimensional data. For other ML fields, a large data set with consistent quality, such as the ImageNet^47^ for computer vision, are fundamental because they provide easily accessible benchmarks. The recent development of the Open Catalysis Project (OCP) data sets serves as an excellent illustration of the benefits derived from large-scale, labeled data sets.^15^ Model training and testing become more convenient and standardized for the entire computational chemistry and materials science community, thanks to the consistent and high-quality data provided by the OCP data set. Although data sets such as the Polymer Gas Separation Membrane Database by the Membrane Society of Australasia^48^ (MSA) and the Open Membrane Databse^7^ (OMD) have been published as solutions, there is still room for improvement in terms of the consistency of labels and experimental conditions. Admittedly, building a large-scale high-quality data set can be costly, but it will definitely be beneficial for both ML model training and evaluation for membrane design.

The second challenge is the choice of the featurization method for membranes. Among the works we reviewed, different featurization methods are used for both polymeric membranes (e.g., *bag-of-fragment*,^22^ cheminformatics fingerprint^18^) and 2D membranes (e.g., crystal structure,^44^ image^27^). The choice of featurization methods can depend on the objective of ML training and can affect the choice of the ML model. Determining the best featurization method of both polymeric and 2D membranes is a comparatively more straightforward task but still requires further benchmarks. Specifically, the challenge in featurizing polymeric membranes stems from their unknown structures, necessitating methods that can accurately determine these structures for effective featurization.^49^

The third challenge is the multiobjective nature of applying ML to membrane design. When designing membranes for specific applications, one should consider not only the performance of the membrane but also properties such as synthesizability and stability. Moreover, for applications like gas separation, one may desire the membrane to be selective to multiple types of gases, rendering the ML membrane design a multiobjective optimization problem. This is a relatively less explored area in ML for membrane design and thus an intriguing future opportunity.

Resolving these challenges enhances the efficacy of ML-assisted membrane design. Moreover, combining ML-assisted design with automated laboratory techniques paves the way for the practical acceleration of material discovery. This effective synergy is effectively demonstrated through the recent advancement that has bridged AI and chemistry experiments by designing workflow to allow a large language model to autonomously design, plan, and perform complex experiments.^50^ Such integration of AI to computational and experimental scientific discovery also promises significant advancements in the membrane field, encompassing applications in water purification, gas separation, biomedical devices, and renewable energy technologies. Collaborative efforts among researchers in materials science, data science, and automation engineering will be essential in realizing the full potential of this approach.

## References

[ref1] Sholl D. S.; Lively R. P. Seven chemical separations to change the world. Nature 2016, 532, 435–437. 10.1038/532435a. 27121824

[ref2] Schrier J. Carbon dioxide separation with a two-dimensional polymer membrane. ACS Appl. Mater. Interfaces 2012, 4, 3745–3752. 10.1021/am300867d. 22734516

[ref3] Lee C.; Wei X.; Kysar J. W.; Hone J. Measurement of the elastic properties and intrinsic strength of monolayer graphene. science 2008, 321, 385–388. 10.1126/science.1157996. 18635798

[ref4] Heiranian M.; Farimani A. B.; Aluru N. R. Water desalination with a single-layer MoS2 nanopore. Nat. Commun. 2015, 6, 8616 10.1038/ncomms9616. 26465062 PMC4634321

[ref5] Cohen-Tanugi D.; Grossman J. C. Water desalination across nanoporous graphene. Nano Lett. 2012, 12, 3602–3608. 10.1021/nl3012853. 22668008

[ref6] Tayyebi A.; Alshami A. S.; Yu X.; Kolodka E. Can machine learning methods guide gas separation membranes fabrication?. Journal of Membrane Science Letters 2022, 2, 100033 10.1016/j.memlet.2022.100033.

[ref7] Ritt C. L.; Stassin T.; Davenport D. M.; DuChanois R. M.; Nulens I.; Yang Z.; Ben-Zvi A.; Segev-Mark N.; Elimelech M.; Tang C. Y.; et al. The open membrane database: Synthesis–structure–performance relationships of reverse osmosis membranes. J. Membr. Sci. 2022, 641, 119927 10.1016/j.memsci.2021.119927.

[ref8] Cao Z.; Liu V.; Barati Farimani A. Why is single-layer MoS2 a more energy efficient membrane for water desalination?. ACS Energy Letters 2020, 5, 2217–2222. 10.1021/acsenergylett.0c00923.

[ref9] Priya P.; Nguyen T. C.; Saxena A.; Aluru N. R. Machine learning assisted screening of two-dimensional materials for water desalination. ACS Nano 2022, 16, 1929–1939. 10.1021/acsnano.1c05345. 35043618

[ref10] Cao Z.; Markey G.; Barati Farimani A. Ozark graphene nanopore for efficient water desalination. J. Phys. Chem. B 2021, 125, 11256–11263. 10.1021/acs.jpcb.1c06327. 34591487

[ref11] Sarvestani A. B.; Chogani A.; Shariat M.; Moosavi A.; Kariminasab H. The effect of nanopores geometry on desalination of single-layer graphene-based membranes: A molecular dynamics study. J. Mol. Liq. 2021, 339, 116749 10.1016/j.molliq.2021.116749.

[ref12] Thomas S.; Silmore K. S.; Sharma P.; Govind Rajan A. Enumerating Stable Nanopores in Graphene and Their Geometrical Properties Using the Combinatorics of Hexagonal Lattices. J. Chem. Inf. Model. 2023, 63, 870–881. 10.1021/acs.jcim.2c01306. 36638043

[ref13] Xie T.; Grossman J. C. Crystal graph convolutional neural networks for an accurate and interpretable prediction of material properties. Physical review letters 2018, 120, 145301 10.1103/PhysRevLett.120.145301. 29694125

[ref14] Ock J.; Guntuboina C.; Barati Farimani A. Catalyst Energy Prediction with CatBERTa: Unveiling Feature Exploration Strategies through Large Language Models. ACS Catal. 2023, 13, 16032–16044. 10.1021/acscatal.3c04956.

[ref15] Chanussot L.; et al. Open Catalyst 2020 (OC20) Dataset and Community Challenges. ACS Catal. 2021, 11, 6059–6072. 10.1021/acscatal.0c04525.

[ref16] Hasnaoui H.; Krea M.; Roizard D. Neural networks for the prediction of polymer permeability to gases. J. Membr. Sci. 2017, 541, 541–549. 10.1016/j.memsci.2017.07.031.

[ref17] Brown K. A.; Brittman S.; Maccaferri N.; Jariwala D.; Celano U. Machine Learning in Nanoscience: Big Data at Small Scales. Nano Lett. 2020, 20 (2–10), 2 10.1021/acs.nanolett.9b04090. 31804080

[ref18] Barnett J. W.; Bilchak C. R.; Wang Y.; Benicewicz B. C.; Murdock L. A.; Bereau T.; Kumar S. K. Designing exceptional gas-separation polymer membranes using machine learning. Science advances 2020, 6, eaaz4301 10.1126/sciadv.aaz4301. 32440545 PMC7228755

[ref19] Zhang Z.; Luo Y.; Peng H.; Chen Y.; Liao R.-Z.; Zhao Q. Deep spatial representation learning of polyamide nanofiltration membranes. J. Membr. Sci. 2021, 620, 118910 10.1016/j.memsci.2020.118910.

[ref20] Fetanat M.; Keshtiara M.; Low Z.-X.; Keyikoglu R.; Khataee A.; Orooji Y.; Chen V.; Leslie G.; Razmjou A. Machine learning for advanced design of nanocomposite ultrafiltration membranes. Ind. Eng. Chem. Res. 2021, 60, 5236–5250. 10.1021/acs.iecr.0c05446.

[ref21] Wang M.; Xu Q.; Tang H.; Jiang J. Machine learning-enabled prediction and high-throughput screening of polymer membranes for pervaporation separation. ACS Appl. Mater. Interfaces 2022, 14, 8427–8436. 10.1021/acsami.1c22886. 35113512

[ref22] Xu Q.; Jiang J. Machine learning for polymer swelling in liquids. ACS Applied Polymer Materials 2020, 2, 3576–3586. 10.1021/acsapm.0c00586.

[ref23] Kumar N.; Salehiyan R.; Chauke V.; Botlhoko O. J.; Setshedi K.; Scriba M.; Masukume M.; Ray S. S. Top-down synthesis of graphene: A comprehensive review. FlatChem. 2021, 27, 100224 10.1016/j.flatc.2021.100224.

[ref24] Russo C. J.; Golovchenko J. A. Atom-by-atom nucleation and growth of graphene nanopores. Proc. Natl. Acad. Sci. U. S. A. 2012, 109, 5953–5957. 10.1073/pnas.1119827109. 22492975 PMC3340994

[ref25] Chen T.; Guestrin C. Xgboost: A scalable tree boosting system. Proceedings of the 22nd acm sigkdd international conference on knowledge discovery and data mining, 2016; pp 785–794.

[ref26] Govind Rajan A.; Silmore K. S.; Swett J.; Robertson A. W.; Warner J. H.; Blankschtein D.; Strano M. S. Addressing the isomer cataloguing problem for nanopores in two-dimensional materials. Nature materials 2019, 18, 129–135. 10.1038/s41563-018-0258-3. 30643239

[ref27] Wang Y.; Cao Z.; Barati Farimani A. Efficient water desalination with graphene nanopores obtained using artificial intelligence. npj 2D Materials and Applications 2021, 5, 66 10.1038/s41699-021-00246-9.

[ref28] He K.; Zhang X.; Ren S.; Sun J. Deep residual learning for image recognition. Proceedings of the IEEE conference on computer vision and pattern recognition, 2016; pp 770–778.

[ref29] Sheshanarayana R.; Govind Rajan A. Tailoring nanoporous graphene via machine learning: Predicting probabilities and formation times of arbitrary nanopore shapes. J. Chem. Phys. 2022, 156, 204703 10.1063/5.0089469. 35649838

[ref30] Prokhorenkova L.; Gusev G.; Vorobev A.; Dorogush A. V.; Gulin A. CatBoost: unbiased boosting with categorical features. Proceedings of the 32nd International Conference on Neural Information Processing Systems 2018, 6639–6649.

[ref31] Adadi A.; Berrada M. Peeking inside the black-box: a survey on explainable artificial intelligence (XAI). IEEE access 2018, 6, 52138–52160. 10.1109/ACCESS.2018.2870052.

[ref32] Molnar C.; Casalicchio G.; Bischl B. Interpretable machine learning–a brief history, state-of-the-art and challenges. Joint European conference on machine learning and knowledge discovery in databases. 2020; pp 417–431.

[ref33] Yang J.; Tao L.; He J.; McCutcheon J. R.; Li Y. Machine learning enables interpretable discovery of innovative polymers for gas separation membranes. Science Advances 2022, 8, eabn9545 10.1126/sciadv.abn9545. 35857839 PMC9299556

[ref34] Shapley L. S. A Value for n-Person Games; Princeton University Press: 1953.

[ref35] Lundberg S. M.; Lee S.-I. A unified approach to interpreting model predictions. Proceedings of the 31st International Conference on Neural Information Processing Systems 2017, 4768–4777.

[ref36] Gao H.; Zhong S.; Dangayach R.; Chen Y. Understanding and Designing a High-Performance Ultrafiltration Membrane Using Machine Learning. Environ. Sci. Technol. 2023, 57, 17831–17840. 10.1021/acs.est.2c05404. 36790106 PMC10666290

[ref37] Jeong N.; Epsztein R.; Wang R.; Park S.; Lin S.; Tong T. Exploring the Knowledge Attained by Machine Learning on Ion Transport across Polyamide Membranes Using Explainable Artificial Intelligence. Environ. Sci. Technol. 2023, 57, 17851–17862. 10.1021/acs.est.2c08384. 36917705

[ref38] Ritt C. L.; Liu M.; Pham T. A.; Epsztein R.; Kulik H. J.; Elimelech M. Machine learning reveals key ion selectivity mechanisms in polymeric membranes with subnanometer pores. Science advances 2022, 8, eabl5771 10.1126/sciadv.abl5771. 35030018 PMC8759746

[ref39] Yeo C. S. H.; Xie Q.; Wang X.; Zhang S. Understanding and optimization of thin film nanocomposite membranes for reverse osmosis with machine learning. J. Membr. Sci. 2020, 606, 118135 10.1016/j.memsci.2020.118135.

[ref40] Gao H.; Zhong S.; Zhang W.; Igou T.; Berger E.; Reid E.; Zhao Y.; Lambeth D.; Gan L.; Afolabi M. A.; et al. Revolutionizing membrane design using machine learning-bayesian optimization. Environ. Sci. Technol. 2022, 56, 2572–2581. 10.1021/acs.est.1c04373. 34968041

[ref41] Deshwal A.; Simon C. M.; Doppa J. R. Bayesian optimization of nanoporous materials. Molecular Systems Design & Engineering 2021, 6, 1066–1086. 10.1039/D1ME00093D.

[ref42] Yaghi O. M. Reticular chemistry in all dimensions. ACS Cent. Sci. 2019, 5, 1295 10.1021/acscentsci.9b00750. 31673626 PMC6820066

[ref43] Wilmer C. E.; Leaf M.; Lee C. Y.; Farha O. K.; Hauser B. G.; Hupp J. T.; Snurr R. Q. Large-scale screening of hypothetical metal–organic frameworks. Nature Chem. 2012, 4, 83–89. 10.1038/nchem.1192. 22270624

[ref44] Wang R.; Zhong Y.; Bi L.; Yang M.; Xu D. Accelerating discovery of metal–organic frameworks for methane adsorption with hierarchical screening and deep learning. ACS Appl. Mater. Interfaces 2020, 12, 52797–52807. 10.1021/acsami.0c16516. 33175490

[ref45] Cao Z.; Magar R.; Wang Y.; Barati Farimani A. Moformer: self-supervised transformer model for metal–organic framework property prediction. J. Am. Chem. Soc. 2023, 145, 2958–2967. 10.1021/jacs.2c11420. 36706365 PMC10041520

[ref46] Kang Y.; Park H.; Smit B.; Kim J. A multi-modal pre-training transformer for universal transfer learning in metal–organic frameworks. Nature Machine Intelligence 2023, 5, 309–318. 10.1038/s42256-023-00628-2.

[ref47] Deng J.; Dong W.; Socher R.; Li L.-J.; Li K.; Fei-Fei L. Imagenet: A large-scale hierarchical image database. 2009 IEEE conference on computer vision and pattern recognition. 2009; pp 248–255.

[ref48] Thornton A.; BD F.; Robeson L. Polymer Gas Separation Membrane Database, 2012.

[ref49] Kong X.; Liu J. An atomistic simulation study on POC/PIM mixed-matrix membranes for gas separation. J. Phys. Chem. C 2019, 123, 15113–15121. 10.1021/acs.jpcc.9b03318.

[ref50] Boiko D. A.; MacKnight R.; Kline B.; Gomes G. Autonomous chemical research with large language models. Nature 2023, 624, 570–578. 10.1038/s41586-023-06792-0. 38123806 PMC10733136

